# Hyperinsulinemia does not cause de novo capillary recruitment in rat skeletal muscle

**DOI:** 10.1111/micc.12593

**Published:** 2019-10-12

**Authors:** Thorbjorn Akerstrom, Daniel Goldman, Franciska Nilsson, Stephanie L. Milkovich, Graham M. Fraser, Christian Lehn Brand, Ylva Hellsten, Christopher G. Ellis

**Affiliations:** ^1^ Department of Nutrition, Exercise and Sports Section of Integrative Physiology University of Copenhagen Copenhagen Denmark; ^2^ Department of Medical Biophysics Schulich School of Medicine & Dentistry University of Western Ontario London Canada; ^3^ Division of BioMedical Sciences Faculty of Medicine Memorial University of Newfoundland St. John's Canada; ^4^ Clamp Competency Centre Novo Nordisk A/S Maaloev Denmark

**Keywords:** insulin, microcirculation, skeletal muscle

## Abstract

**Objective:**

The effect of insulin on blood flow distribution within muscle microvasculature has been suggested to be important for glucose metabolism. However, the “capillary recruitment” hypothesis is still controversial and relies on studies using indirect contrast‐enhanced ultrasound (CEU) methods.

**Methods:**

We studied how hyperinsulinemia effects capillary blood flow in rat extensor digitorum longus (EDL) muscle during euglycemic hyperinsulinemic clamp using intravital video microscopy (IVVM). Additionally, we modeled blood flow and microbubble distribution within the vascular tree under conditions observed during euglycemic hyperinsulinemic clamp experiments.

**Results:**

Euglycemic hyperinsulinemia caused an increase in erythrocyte (80 ± 25%, *P* < .01) and plasma (53 ± 12%, *P* < .01) flow in rat EDL microvasculature. We found no evidence of de novo capillary recruitment within, or among, capillary networks supplied by different terminal arterioles; however, erythrocyte flow became slightly more homogenous. Our computational model predicts that a decrease in asymmetry at arteriolar bifurcations causes redistribution of microbubble flow among capillaries already perfused with erythrocytes and plasma, resulting in 25% more microbubbles flowing through capillaries.

**Conclusions:**

Our model suggests increase in CEU signal during hyperinsulinemia reflects a redistribution of arteriolar flow and not de novo capillary recruitment. IVVM experiments support this prediction showing increases in erythrocyte and plasma flow and not capillary recruitment.

**Abbreviations**
BN: simulated baseline
CEU: contrast‐enhanced ultrasound
D: vessel inner diameter
EDL: extensor digitorum longus
FOV: fields of view
GIR: glucose infusion rate
IVVM: intravital video microscopy
RBC: red blood cell, erythrocyte
SHI: simulated hyperinsulinmemia

## 1 INTRODUCTION

The skeletal muscle vasculature delivers insulin and glucose to muscle cells, and skeletal muscle capillarization is an important contributing factor to insulin sensitivity.^1^ Blood flow distribution within the muscle microvasculature might therefore be important for glucose metabolism. Over the last two decades, a number of studies have suggested that insulin affects the distribution of blood flow within the muscle microvasculature^2^, ^3^, ^4^, ^5^, ^6^, ^7^ It is believed that insulin increases the number of capillaries that receive blood flow and thereby enhances the delivery of glucose and insulin to the myocyte. This phenomenon has been termed “capillary recruitment.” Whether capillary recruitment actually occurs has frequently been debated.^8^, ^9^, ^10^, ^11^

The proponents of the capillary recruitment hypothesis rely mainly on indirect data collected using contrast‐enhanced ultrasound (CEU).^2^, ^3^, ^5^, ^6^, ^7^ Opponents of the capillary recruitment hypothesis tend to point to direct observation of the microvasculature using intravital video microscopy (IVVM). Indeed, most^12^, ^13^, ^14^, ^15^, ^16^, ^17^, ^18^, ^19^ but not all^20^, ^21^ IVVM studies show that 80%‐95% of all capillaries have red blood cells or plasma flowing through them at baseline and that there is no capillary recruitment of additional vessels with contraction^14^, ^16^, ^19^ or vasodilator stimulation.^17^

IVVM techniques have been criticized for possibly creating a hyperemic state that is unable to exhibit capillary recruitment in response to normal physiological stimuli due to the potential for surgical trauma or the use of superfusate solutions. However, there is strong evidence that IVVM preparations do reflect the normal physiological response to stimuli since these preparations have been instrumental in uncovering fundamental mechanisms of microvascular blood flow regulation (Segal;^22^ Murrant and Sarelius^23^). Although IVVM does not typically show “de novo” capillary recruitment, that is, a reserve of capillaries with no red blood cell or plasma flow that can be recruited when needed, IVVM does show a more uniform distribution of RBCs among these perfused capillaries which reduces the heterogeneity of capillary hematocrits and supply rates. This network flow redistribution is primarily governed by rheological factors that passively occur in response to an increase in blood flow, not to the active “opening” of new capillary flow paths.^24^

Some have also proposed that the use of thin muscle preparations such as the spinotrapezius or cremaster might not be representative of larger muscles involved in locomotion or lifting.^8^, ^9^ For this study, we chose to use the extensor digitorum longus (EDL) muscle, a relatively thick (>1 mm), locomotor muscle in the rat hind limb that can be surgically reflected without damage to the muscle belly or disruption of tissue blood flow, and minimal manipulation of surrounding tissues.^19^ The available literature also suggests that surgical exteriorization does not affect vascular function.^25^, ^26^

We also have extensive experience with this IVVM preparation of the EDL muscle. Under baseline conditions, 85%‐90% of capillaries containing red blood cells (RBC) are continuously perfused with RBCs. The remaining 10%‐15% have either intermittent or stopped RBCs flow. Capillaries with plasma flow only cannot be consistently detected with our microscopy setup, nor can we detect “closed” capillaries if they exist. Perfused capillaries have a mean RBC velocity, RBC supply, and hemoglobin O_2_ saturation of 0.150 mm/s, 7.5 RBC s^−1^, and 65%, respectively.^13^ These results are consistent with a healthy microvascular bed at physiological baseline conditions and should therefore serve as a good model for testing the effect of insulin on the microcirculation. Thus, the primary objective of the present study was to perform euglycemic hyperinsulinemic clamp experiments on rats for the first time combined with IVVM analysis of the microcirculation in the EDL muscle to test whether hyperinsulinemia leads to capillary recruitment and redistribution of microvascular blood flow in skeletal muscle. Blood flow was determined by measuring capillary RBC velocity, hematocrit, and diameter, and using these values to calculate RBC, plasma and blood flow rates applying established relationships. Since glucose and insulin are carried in the plasma, we were thus able to determine whether there was evidence of their redistribution with hyperinsulinemia.

We also defined a secondary objective, which was to attempt to reconcile CEU and IVVM results.

The increase in ultrasound signal with microbubbles during hyperinsulinemia may be explained without de novo capillary recruitment in a way that is consistent with IVVM data. The size of the rat EDL muscle makes it impractical to make a direct comparison between the two techniques, and our previous attempts to visualize fluorescently labeled microbubbles in the EDL were unsuccessful, likely due to the low density of microbubbles relative to RBCs. Hence, we chose to develop a mathematical model of blood flow and microbubble distribution within the microvasculature. Although microbubbles have been proposed to distribute in a manner similar to erythrocytes,^27^ it is also possible that they are more sensitive to the flow distribution at bifurcations than erythrocytes due to their rigidity.^28^ This would account for the enrichment of microbubbles relative to erythrocytes in some flow paths as reported by Lindner and co‐workers.^27^ Our mathematical model examines the effect of a redistribution of blood flow on microbubble distribution to investigate whether it is possible to reconcile CEU and IVVM results.

## 2 RESEARCH DESIGN AND METHODS

### 2.1 Animals

Intravital video sequences of capillary networks in the EDL muscle of eight rats at basal conditions and during a hyperinsulinemic euglycemic clamp were used as the basis for the present work. Animal protocols were approved by the Animal Care and Use Committee of the University of Western Ontario. Male Sprague Dawley rats, seven weeks of age (n = 8; Charles River), were housed in dedicated animal quarters at the University of Western Ontario with free access to food (Purina LabDiet RMH 3000) and water on a 12/12‐h light/dark cycle. The rats were acclimatized for 1 week after delivery. On the day of the experiment, the rats weighed 165 ± 4 g.

### 2.2 Surgical preparation of rats

The animals were anesthetized with an intraperitoneal, IP, bolus of α‐chloralose (80 mg/kg, Fluka Analytical #23 120‐100 g), and urethane (500 mg/kg, Sigma‐Aldrich U2500‐500g) in saline. All rats were instrumented with catheters (Tygon S‐54‐HL Microbore Tubing, inner diameter: 0.41 mm, Norton Performance Plastics) in the right jugular vein (for infusion of insulin, glucose, and anesthesia) and left carotid artery (for blood sampling and blood pressure measurements) under aseptic conditions. Tracheotomy was performed, and a tracheotomy tube (plastic end of IV catheter, 18 G) was inserted to facilitate spontaneous ventilation.

Throughout the experiment, the animal was kept anesthetized by infusing a mixture of α‐chloralose (80 mg/kg, Fluka Analytical #23 120‐100 g) and urethane (500 mg/kg, Sigma‐Aldrich U2500‐500g) in saline at a rate of 0.8‐1.2 mL/h. Depth of anesthesia was assessed by continuous monitoring of mean arterial blood pressure which was kept within a range of 90‐100 mm Hg by varying the infusion rate of the anesthetic.

The EDL muscle, a bellied muscle in the hind limb, was chosen for this study since the muscle is easily exposed with minimal trauma and the collagen sheath covering the muscle is very thin, permitting good visualization of the microcirculation. The EDL muscle was prepared for in vivo microscopy using blunt dissection and externalized as previously described.^19^ Briefly, a small section of the skin was removed from the lateral side of the right lower hind limb, exposing the fascia capsule of the underlying muscles. Superficial dissection of the capsule and blunt separation of the surrounding muscles allowed the EDL to be isolated. Silk ligature was threaded under the intact muscle and secured with a square knot on the distal portion of the EDL tendon. The tendon was then severed between the ligature and muscle insertion, leaving the ligature securely attached to the free end of the EDL tendon. After EDL dissection, the animal was transferred to the microscope stage and placed on its right side in a semi‐prone position. The ligature secured to the EDL tendon was then taped to the stage such that the lateral side of the muscle was facing the objectives and the muscle maintained a length approximate to the resting position in vivo. The muscle was moistened with 37°C saline and covered on the medial side with a small square of plastic film (~2 × 2 cm, polyvinylidene chloride, Saran) and a glass coverslip to isolate the muscle from the external environment and ensure that the microvasculature is the only O_2_ source for the tissue.

### 2.3 Baseline and hyperinsulinemic euglycemic clamp measurements

Immediately following the surgical preparation, the animal rested on the microscope stage for 30 minutes prior to the start of the experiment. This pre‐clamp period consisted of a 15‐minute infusion of anesthesia followed by 15‐minute infusion of anesthesia plus saline. The experiment started with infusion of saline (~0.6 mL/h, Baxter Canada) for 30 minutes with arterial blood samples drawn every 5 minutes as described below. The volume of the saline infusion matched the combined insulin and glucose infusion during the last 30 minutes of the hyperinsulinemic euglycemic clamp.

Immediately following the baseline measurements, a bolus (300 ρmol/kg) of recombinant human insulin (Actrapid, Novo Nordisk) diluted to 1500 pmol/L in a buffer (pH 7.4) consisting of 140 mmol/L NaCl, 5 mmol/L Na_2_HPO_4_, and, in order to prevent protein adsorption, 70 ppm Tween20 was infused over 2 minutes followed by a constant rate (30 ρmol/kg/min) infusion for 70 minutes. A 40% glucose solution (400 mg/mL, pH 7.4, VWR International) was infused at variable rates (GIR) to maintain euglycemia.

Arterial blood was sampled (10 µL) and glucose concentration measured at 5‐minutes intervals (HemoCue Glucose 201 RT, HemoCue AB) throughout the experiment. The average GIR during the final 30 minutes of the hyperinsulinemic euglycemic clamp, at which time steady state was reached, was used as a measure of insulin sensitivity.

Blood was drawn at 0 minutes (200 µL), 30 minutes (200 µL, end of baseline), 70 minutes, and 100 minutes (200 µL, steady‐state portion of clamp) for determination of plasma C‐peptide and insulin. Blood was centrifuged (5 minutes, 13 000 *g*, 4°C), and plasma was stored at −20°C until further analysis. At 30 and 100 minutes, an arterial blood sample was drawn into a capillary tube and centrifuged to measure systemic hematocrit. At the end of the experiment (100 minutes), an additional 95 µL of blood was collected for analysis of arterial pH, pCO_2,_ and pO_2_ using a point of care device (iSTAT with CG4 + cartridge, Abbott). The hemoglobin oxygen saturation (sO_2_) was calculated using the model proposed by Dash and Bassingthwaighte.^29^

A pressure transducer connected to the carotid cannula allowed heart rate and mean arterial pressure to be monitored (Digi‐Med System, Micro‐Med) and recorded with an attached computer. Body temperature was continuously monitored using a rectal probe. At the end of the experiment, all animals were euthanized with an overdose of pentobarbital (110 mg/kg, IV).

### 2.4 Dual spectrophotometric intravital video microscopy

The muscle was transilluminated with a 75‐W Xenon lamp and viewed through a Olympus IX‐81 inverted microscope equipped with X10 and X20 objectives and a DualCam (parfocal beam splitter) fitted with 442 nm and 454 nm interference filters (10 nm bandpass) for absorption spectroscopy measurement of hemoglobin oxygen saturation. Simultaneous frame‐by‐frame video was captured at each wavelength using two identical Rolera XR digital video cameras streaming video sequences (696 X 520, 21 frames s^‐1^) directly to a single acquisition computer using custom capture software (Neovision, Czech Republic). The two cameras were temporally synchronized and aligned such that video images were in register. Video sequences of capillary networks were acquired from the same ten 20X fields of view (FOV) at baseline with saline infusion and 45 minutes following initiation of the hyperinsulinemic euglycemic clamp. Each FOV was captured for one minute (1260 video frames for each camera). The total acquisition time from capture of the first FOV to the last was twenty to thirty minutes. The same two overlapping 10X FOV were also acquired at baseline with saline infusion and 20 minutes after starting hyperinsulinemic euglycemic clamp. In all cases, each FOV was acquired for one minute (1260 frames) and the acquisition time for both was less than five minutes.

### 2.5 Microvascular measurements

Hemodynamic measurements were made offline from video sequences of individual, in‐focus capillaries within each field of view. Automated measurements for RBC velocity (mm/s), lineal density (RBC mm^−1^), and supply rate (RBC s^−1^) were made on a frame‐by‐frame basis from each 60s sequence using custom analysis software described elsewhere.^13^, ^30^, ^31^ Briefly, segments of in‐focus capillaries within each FOV were selected and outlined from functional images generated by processing the intravital video sequences.^32^ Functional images provide high contrast delineation between tissue and the red blood cell column (luminal space swept out by the passage of RBCs) and were used to determine vessel diameter and segment length. Functional capillary density in the EDL muscle was determined using the method described by Ellis et al^33^ A transparency with three 150 µm horizontal staggered reference lines drawn perpendicular to the muscle fibers was placed over the computer screen during playback of the 20X videos, and capillaries with clearly distinguishable RBCs were counted. Capillaries that intersected the reference line were classified over a 30‐s time interval as being either (a) continuous, that is, a continuous uninterrupted flow of RBCs, (b) intermittent, that is, RBC flow either stopped for less than 30 seconds at least once or reversed during the observation period, or (c) stopped, that is, RBCs were stationary for 30 seconds. Functional capillary density was calculated as the number of capillaries per millimeter test line. Space‐time images were generated for each in‐focus vessel in the field of view, displaying the light intensity along the centerline of a vessel over time and hence the passage of RBCs separated by plasma through the segment. Automated routines were used to distinguish RBCs from plasma in order to measure RBC lineal density and to quantify the spatial displacement of the RBC column from frame‐to‐frame to measure the RBC velocity. Capillary (tube) hematocrit for each video frame was calculated as the product of lineal density and RBC volume divided by the volume of the vessel segment. Vessel segment volume was calculated using mean vessel diameter over the segment length selected and by assuming the vessel had a circular cross section. Frame‐by‐frame RBC supply rate (RBC s^−1^) was calculated as the product of the RBC velocity (mm/s) and RBC lineal density (RBC mm^−1^). RBC oxygen saturations were determined from the ratio of RBC optical density at the two wavelengths based on an in vivo calibration.^34^ Optical density was determined using the Lambert‐Beer law, OD = log (*I*_O_/*I*_RBC_) where I_O_ is the plasma light intensity and *I*_RBC_ is the RBC light intensity obtained from the space‐time image.

The *volumetric flow rates* of erythrocytes, plasma, and blood (ρL/s) were calculated from the measured hemodynamic data above. RBC flow was calculated simply as the RBC supply rate (RBCs s^−1^) multiplied by the mean RBC volume for rat (65 μm^3^;^35^). Since we are unable to measure plasma velocity directly, plasma and blood flow rates were estimated using a published relationship for the Fahraeus effect.^36^ This relationship uses the measured capillary (tube) hematocrit and capillary diameter to calculate the discharge hematocrit (discharge hematocrit is the hematocrit one would measure if the blood flowing through the vessel was collected in a reservoir). Blood velocity was calculated as the ratio of measured capillary (tube) hematocrit divided by calculated discharge hematocrit times the measured RBC velocity. Plasma velocity was calculated from the weighted blood and RBC velocity based on the capillary hematocrit. Blood and plasma flows were calculated assuming a circular cross section for each capillary.

The 10X overlapping FOV were analyzed for capillary hemodynamic data within each of the discrete capillary networks. Networks with measurements from at least four capillaries at baseline and 20 minutes following start of insulin infusion resulted in a pooled sample from seven animals of 17 discrete networks with a total of 147 capillaries. One animal was excluded from this analysis, because there were fewer than four capillaries in each capillary network that could be analyzed at both time points.

### 2.6 Plasma protein concentrations

Rat C‐peptide, rat insulin, and human insulin (used for infusion during hyperinsulinemic euglycemic clamp) were measured at Novo Nordisk facilities in Maaloev, Denmark, using in‐house luminescent oxygen channeling immunoassays.^37^ The lower limits of quantification for these assays are 18 pmol/L (Rat C‐peptide), 20 pmol/L (rat insulin), and 15 pmol/L (human insulin in rat plasma).

### 2.7 Modeling

#### 2.7.1 Microvascular network geometry and arteriolar asymmetry at bifurcations

Our baseline microvascular network geometry is based on published data on the structure of arteriolar trees in the rat EDL muscle.^38^ Starting from a first‐order arteriole (1A) with an inner diameter (*D*) of 75 μm, our network bifurcates a number of times (depending on the particular flow path) until reaching terminal arterioles with a mean diameter of ~11 µm. To model the redistribution of flow with hyperinsulinemia, we simulated a modified network with decreased asymmetry of diameters at each bifurcation (average of 5.5% vs 7% in the baseline network) and a 50% increase in total flow. The selection of parameters for the simulated hyperinsulinemia network reflected the IVVM measurements. We sought to simulate the effect this asymmetry would have on both blood and microbubble flow distribution.

#### 2.7.2 Blood flow model

Our steady‐state two‐phase blood flow model^39^ is based on the model originally described by Pries and co‐workers.^36^, ^40^ This model conserves blood and RBC volume flow at each node connecting two or three vessel segments (assumed to be cylindrical) and includes an empirical function describing the non‐linear way in which RBCs distribute at diverging bifurcations (RBC bifurcation law)^41^, ^42^, ^43^, ^44^ under the laminar flow conditions found in the microvasculature. It also includes empirical relations describing how blood viscosity varies as a function of vessel diameter and hematocrit, and how RBC velocity differs from blood velocity in microvessels (RBC Fahraeus effect).^36^ Since RBCs travel closer to the vessel center and have a higher average velocity than blood as a whole, the discharge (flow‐averaged) hematocrit is larger than tube (volume‐averaged) hematocrit. To compute blood and RBC flow in our modeled microvascular network, we assume fixed pressure drops (12 and 18 mm Hg for the baseline network and the simulated hyperinsulinemia network, respectively) between the inlet node (start of 1st order arteriole) and all outlet nodes (ends of terminal arterioles), and we also assume a physiological inflow hematocrit of 0.42 and that all arterioles and capillaries in the model are perfused with erythrocytes and plasma.

#### 2.7.3 Microbubble flow

Encapsulated microbubbles used in CEU imaging are nearly rigid spheres with diameters in the range of ~1‐10 micrometers. 45 Since the ratio of microbubbles to RBCs is typically ~1:6000,^4^ microbubbles travel as isolated particles and are expected to be more concentrated toward the center of microvessels than are RBCs. The more numerous deformable RBCs are more uniformly distributed across the lumen with a reduced concentration near the wall. Although the exact radial distribution of microbubbles is not known, it should depend on the ratio of microbubble diameter to vessel diameter.^46^ However, since the biophysical properties of microbubble distribution in microvessels are not precisely known, we assume a simple model based on the data in Keller et al^28^ Essentially, we assume microbubbles behave like RBCs for arteriolar diameters D > 30 microns, and have a more preferential distribution at diverging bifurcations for D < 30 microns (see Appendix). When combined with our blood flow calculation and conservation of microbubble flow at each node, the assumed microbubble behavior allows us to estimate microbubble flow rates and densities throughout our arteriolar network model. Since it is expected that a greater number of microbubbles will reside in the capillary beds supplied by the arteriolar network, we also model microbubble concentration in the capillaries. Assuming the arteriolar tree supplies approximately 2.2 x 10^4^ capillaries and a typical capillary RBC supply rate is ~12 RBC s^−1^ (Ellis et al, 2002b), the arteriolar network has an RBC supply rate of ~2.6 × 10^5^ RBC s^−1^. For our microbubble flow calculations, we assume the supply rate of microbubbles in the 1st order arteriole is 6000 times smaller than the supply rate of RBCs,^4^ which gives approximately 43 microbubbles s^−1^ for the baseline network. If the capillaries are uniformly distributed across the terminal arterioles and all receive the same proportion of flow from their parent vessels, we can estimate the number of microbubbles in capillaries (see Appendix).

### 2.8 Statistical analyses

We used paired t tests (PROC TTEST) (SAS version 9.4, SAS Institute) to determine whether there were differences between baseline and hyperinsulinemic euglycemic clamp for all experimental parameters (n = 8) except for GIR, HI, and RI. A one‐sample t test (PROC TTEST) was used to compare GIR, HI, and RI to 0. We fit linear regression functions (Prism 5.0, GraphPad Software Inc) to experimental data of percentage change (calculated as ((hyperinsulinemic euglycemic clamp ‐ baseline)/ baseline) × 100) in blood flow and plasma flow during hyperinsulinemia as a function of baseline RBC flow or plasma flow. The fit of the linear regression functions was evaluated using Wald‐Wolfowitz tests.

The distribution of all data was evaluated using probability plots and Kolmogorov‐Smirnov tests. The distribution of difference derived from the paired t test was evaluated using Q‐Q plots. All data were normally distributed. Data are presented as mean ± standard error (SE) or percentage change (calculated as [(mean post‐mean pre)/ mean pre] × 100). Mathematical modeling data are presented as generated by the model. Significance for all tests was set at *P* < .05.

## 3 RESULTS

### 3.1 Characteristics of baseline and hyperinsulinemic euglycemic clamp

Blood pressure and core temperature remained unchanged throughout the experiment (Table 1), whereas systemic hematocrit dropped from 37.5 ± 1.0 to 36.0 ± 1.1% (*P* < .05, Table 1). At the end of the experiment, the systemic arterial pH, pCO_2_, pO_2,_ and sO_2_ were within normal range (Table 1).

**Table 1:** Baseline and hyperinsulinemic euglycemic clamp characteristics

	Baseline	Hyperinsulinemic euglycaemic clamp
MAP (mm Hg)	96 ± 3	93 ± 5
Core temperature (ºC)	37.5 ± 0.7	37.5 ± 0.7
Systemic hematocrit (%)	37.5 ± 1.0	36.0 ± 1.1*
pH	n.m.	7.43 ± 0.02
pCO_2_ (mm Hg)	n.m.	36 ± 2
pO_2_ (mm Hg)	n.m.	78 ± 4
sO_2_ (%)	n.m.	91 ± 1
C‐peptide (ρmol/L)	346 ± 38	170 ± 29*
Rat insulin (ρmol/L)	198 ± 15**	n.m.
Human insulin (ρmol/L)	n.m.	497 ± 29**

Note

Data are presented as mean ± SE. Mean arterial pressure (MAP), core temperature, systemic hematocrit, and C‐peptide were measured at baseline and during the steady‐state portion of the hyperinsulinemic euglycemic clamp (hyperinsulinemic euglycemic clamp). pH, partial pressure of carbon dioxide (pCO_2_), and oxygen (pO_2_) were measured at the end of the hyperinsulinemic euglycemic clamp, but were not measured (n.m.) during baseline to reduce the amount of blood collected. The hemoglobin oxygen saturation (sO_2_) was calculated based on the measured pH, pCO_2,_ and pO_2_ values. Rat insulin and human insulin were measured at baseline and hyperinsulinemic euglycemic clamp, respectively.

* *P* < .05, compared to baseline.

** *P* < .01, compared to 0.

**Figure 1:**
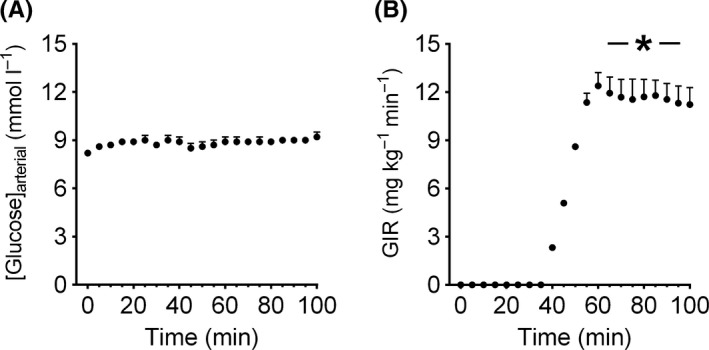
The figure depicts arterial glucose concentration (GIR) (A) and glucose infusion rate (B) at baseline (0‐30 min) and during the hyperinsulinemic euglycemic clamp (35‐100 min) in 7‐week‐old Sprague Dawley rats (n = 8). Blood glucose was maintained at the same concentration during the hyperinsulinemic euglycemic clamp as during baseline (~8.5 mmol/L). GIR during the steady‐state phase (70‐100 min) of the hyperinsulinemic euglycemic clamp was compared to 0. The data are presented as mean ± SE. *: *P* < .01, compared to 0

Arterial glucose concentration did not change throughout the experiment (Figure 1A). At baseline, plasma concentration of rat insulin and C‐peptide was 198 ± 15 ρmol/L and 346 ± 38 ρmol/L, respectively (Table 1). At the onset of the hyperinsulinemic euglycemic clamp, recombinant human insulin was infused and the concentration rose to 497 ± 29 ρmol/L (*P* < .01, Table 1) whereas C‐peptide concentration fell by 51 ± 9% from baseline to the steady‐state phase of the hyperinsulinemic euglycemic clamp (*P* < .01, Table 1). In the same space of time, GIR rose to 12.6 ± 0.4 mg/kg/min (*P* < .01, Figure 1B).

### 3.2 Insulin increases capillary velocities and flow rates

**Figure 2:**
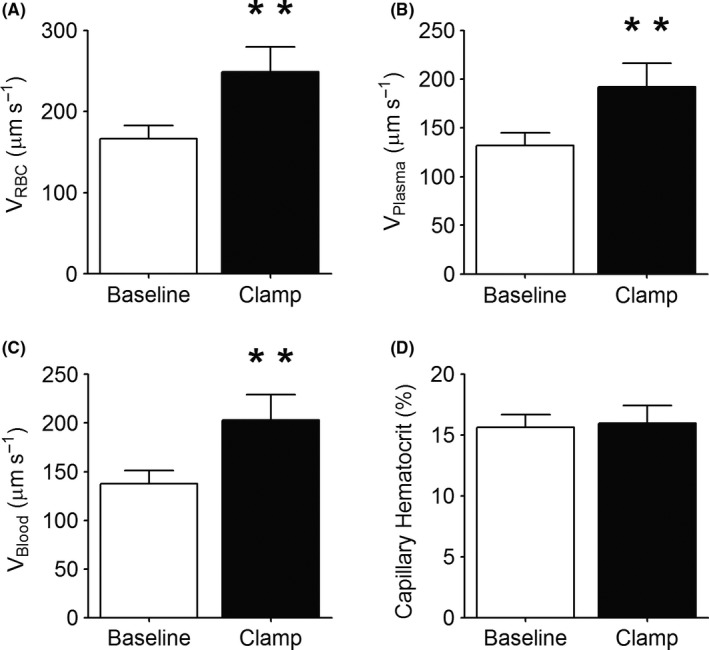
The figure depicts capillary red blood cell (RBC) velocity (*V*_RBC_) (A), plasma velocity (*V*_Plasma_) (B), blood velocity (*V*_Blood_) (C), and capillary hematocrit (D) at baseline and during the steady‐state portion of a hyperinsulinemic euglycemic clamp (Clamp) in the EDL muscle of 7‐week‐old Sprague Dawley rats (n = 8). The data are presented as mean ± SE. **: *P* < .01, compared to baseline

Mean RBC velocity in the observed capillaries was 49 ± 12% greater during the steady‐state portion of the hyperinsulinemic euglycemic clamp compared with baseline (*P* < .01, Figure 2A). In line with this, calculated plasma and blood velocity increased by 47 ± 12% and 48 ± 12%, respectively (*P* < .01, Figure 2B&C), whereas capillary hematocrit remained unchanged (*P* = .58, Figure 2D).

**Figure 3:**
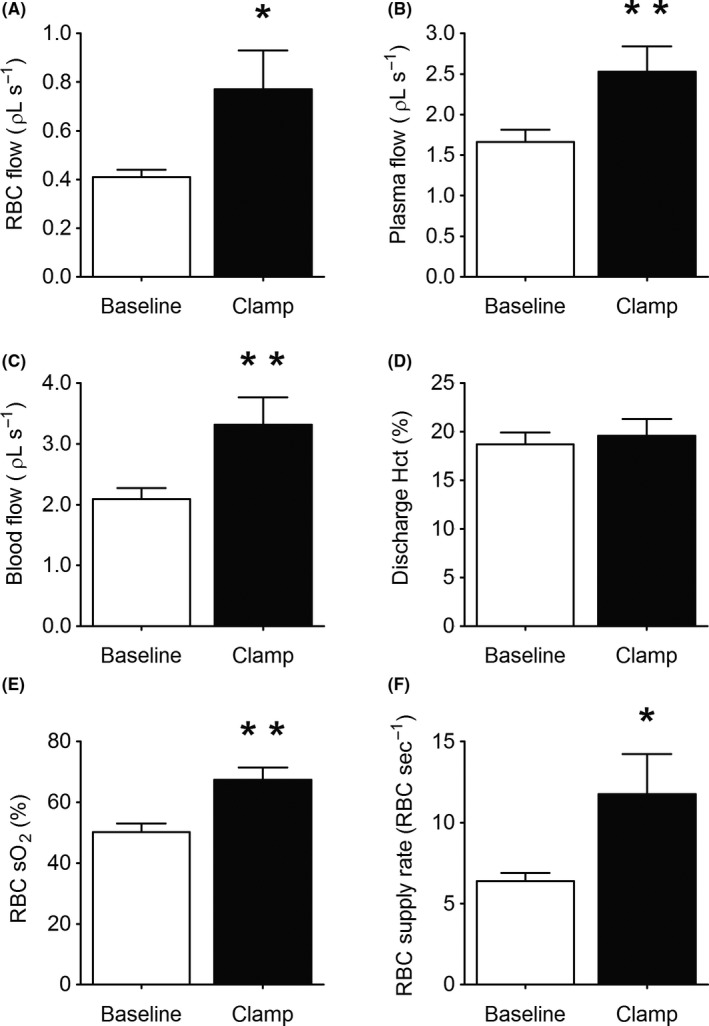
The figure depicts capillary red blood cell (RBC) flow (A), plasma flow (B), blood flow (C), discharge hematocrit (Hct) (D), capillary RBC O_2_ saturation (E), and RBC supply rate (F) at baseline and during the steady‐state portion of a hyperinsulinemic euglycemic clamp (Clamp) in the EDL muscle of 7‐week‐old Sprague Dawley rats (n = 8). The data are presented as mean ± SE. *: *P* < .05, compared to baseline. **: *P* < .01, compared to baseline

Capillary flow rates followed a similar pattern to changes in velocity. RBC and plasma flow increased by 80 ± 25% (*P* < .05, Figure 3A) and 53 ± 12% compared with baseline (*P* < .01, Figure 3B), respectively. Blood flow increased by 58 ± 14% from baseline (*P* < .05, Figure 3C), but there was no change in the calculated discharge hematocrit (*P* = .49, Figure 3D).

### 3.3 Hemoglobin O_2_ saturation of capillary RBCs increases with hyperinsulinemia

The hemoglobin oxygen saturation of the RBCs flowing through the capillaries increased 34% (*P* < .01) from 50 ± 3 to 67 ± 11% (Figure 3E) in line with the increase in RBC supply rate from 6 ± 1 to 12 ± 3 RBCs s^−1^ (*P* < .05, Figure 3F).

### 3.4 Insulin does not increase functional capillary density

**Figure 4:**
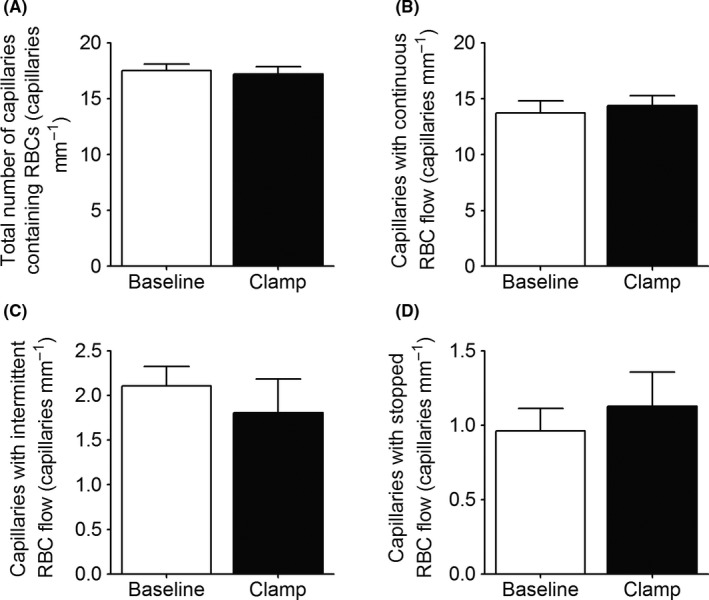
The figure depicts the total number of capillaries containing red blood cells (RBC) (A), the number of capillaries with continuous RBC flow (B), the number of capillaries with intermittent RBC flow (C), and the number of capillaries with stopped RBC flow (D) during baseline and during the steady‐state portion of a hyperinsulinemic euglycemic clamp (Clamp) in the EDL muscle of 7‐week‐old Sprague Dawley rats (n = 8). The data are presented as mean ± SE

**Figure 5:**
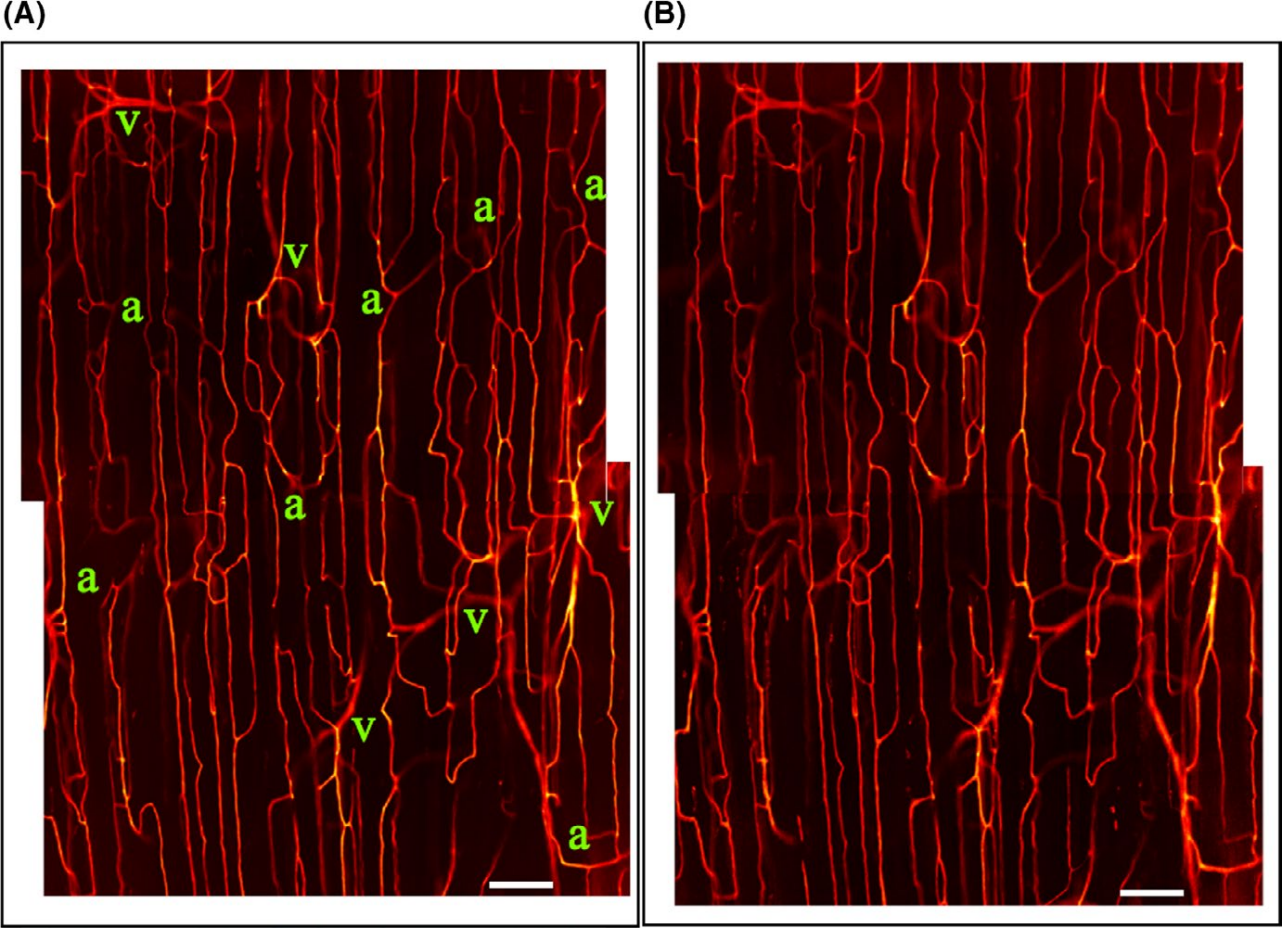
The figure shows representative functional images of the same two overlapping fields of view at baseline (Panel A) and 20 minutes after starting the insulin infusion (Panel B) from one animal. The functional images are produced from the analysis of the one minute video sequence and show only vessels with RBC flow during the one minute. Arterioles (a) penetrate from deeper in the muscle to supply the capillary networks in focus near the muscle surface and venules (v) disappear back into the muscle after collecting flow from the capillaries. Scale bar is 100 μm

Although the mean RBC velocity increased from baseline to the steady‐state portion of the hyperinsulinemic euglycemic clamp, there was no change in the total number of capillaries containing RBCs (*P* = .32, Figure 4A). To allow for a more refined analysis, we distinguished between capillaries with continuous, intermittent, and stopped RBC flow (Figure 4). The clear majority of capillaries viewed in this preparation were capillaries with continuous flow, and this number did not change during the hyperinsulinemic euglycemic clamp compared with baseline (*P* = .49, Figure 4B). Of all capillaries viewed, ~5% had stationary RBCs for 30 seconds (stopped flow) and ~10% had intermittent RBC flow at baseline (Figure 4C and D). There was no change in the number of capillaries with intermittent (*P* = .54) or stopped (*P* = .38) RBC flow between baseline and hyperinsulinemic euglycemic clamp (Figure 4C and D). Consistent with the 20X capillary density data, two representative functional images of overlapping FOV at 10X generated from 1‐minute video sequences at baseline and 20 minutes after starting the insulin infusion show that there was no recruitment of new capillaries with RBC flow (Figure 5 and Suppinfo S1 442‐nmVideos[Link], [Link]). The Supplemental Videos were recorded at 10X magnification at baseline and 20 minutes after initiating hyperinsulinemic euglycemic clamp protocol.

### 3.5 Insulin makes RBC flow more homogenous

**Figure 6:**
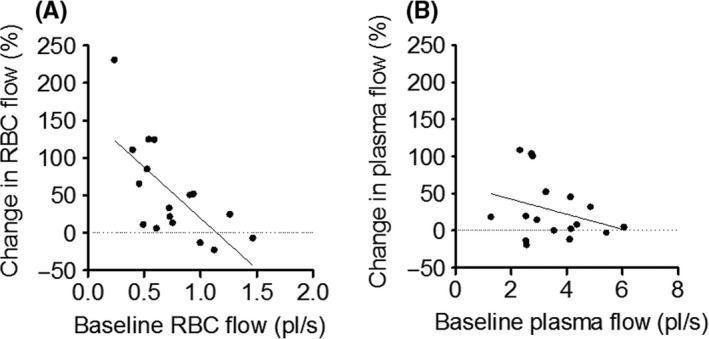
The figure depicts the percent (%) change in red blood cell (RBC) flow (A) and plasma flow (B) from baseline to the steady‐state portion of a hyperinsulinemic euglycemic clamp plotted as a function of baseline RBC flow (pL/s). The data represent pooled samples of 17 discrete networks with a total of 147 capillaries in the EDL muscle of 7‐week‐old Sprague Dawley rats (n = 7). The linear regression function fitted to the RBC flow data is represented by a solid line. The regression function is *y* = 136*x *+ 156 (*P* < .01), and the accompanying r^2^‐value is 0.47

Comparisons of RBC flow using the 10X overlapping FOV revealed an increase in RBC flow from baseline to hyperinsulinemia that was not uniform (Figure 6A). The capillaries with the lowest baseline flows had the largest increases in RBC flow during hyperinsulinemia, whereas RBC flow did not increase or in some instances decreased in the capillaries with the highest baseline flow rates (*P* < .01, Figure 6A). Thus, insulin appeared to result in a redistribution of RBCs that increased the homogeneity of RBC flow in the capillary bed. However, plasma flow increased more uniformly and the increase in plasma flow as a response to hyperinsulinemia was not affected by baseline plasma flow (*P* = .24, Figure 6B). Insulin did not result in a redistribution of plasma flow.

### 3.6 Modeling results

**Figure 7:**
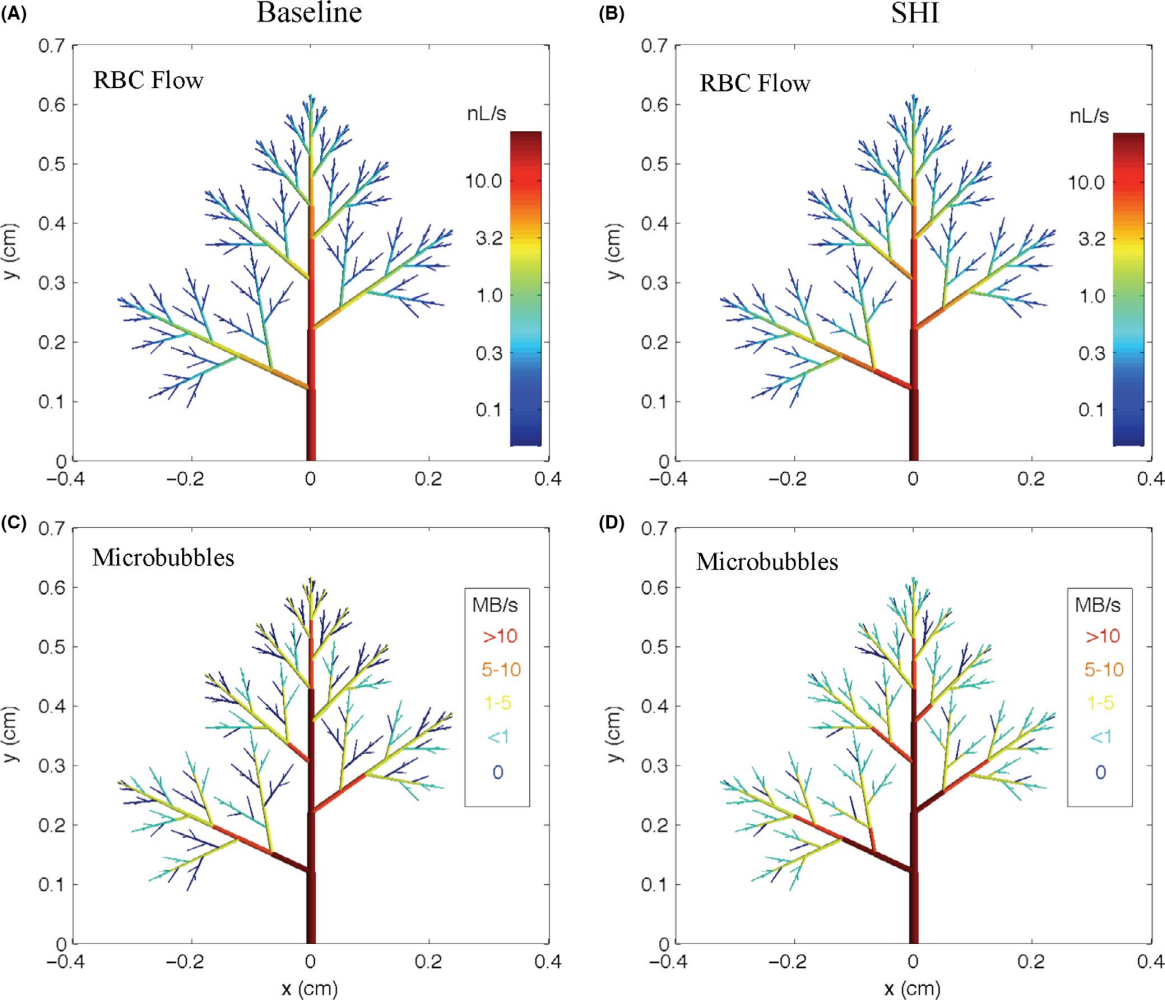
The figure depicts our modeling results for steady‐state red blood cell flow (A&B) and microbubble distribution (C&D) in the simulated baseline (BN) and simulated hyperinsulinemia (SHI) microvascular networks. The y‐ and x‐axes serve as a reference to the scale of the microvascular networks. In panels A and B, RBC flow is represented by a gradient colourmap where red represents high flow and blue represents lower flow. RBC flow (nL/s) is shown on a logarithmic scale to improve flow visualization in the higher order arterioles. The arterioles receiving microbubbles in panels C and D are colored red (>10/s), orange (5‐10/s), yellow (1‐5/s), and light blue (>0/s but < 1/s), while those not receiving any microbubble flow are coloured dark blue

**Figure 8:**
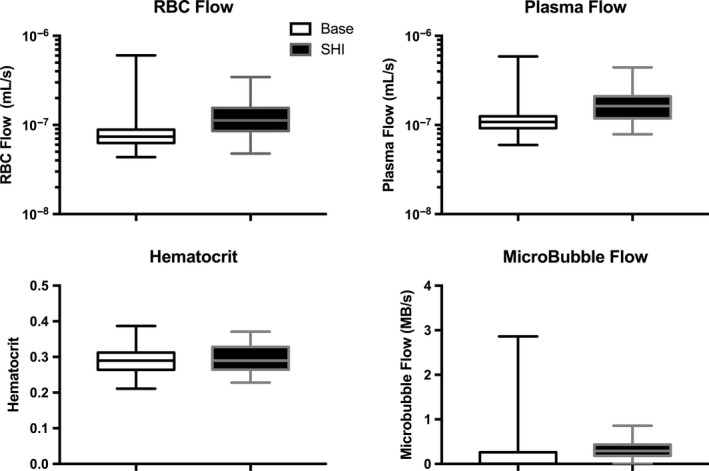
The figure depicts our modeling results in box plots showing the median, first and third quartiles, and maximum and minimum values of RBC flow (A), plasma flow (B), and tube hematocrit (C) in the terminal arterioles for the baseline and simulated hyperinsulinemia (SHI) networks. Panel D depicts modeling results for the distribution of microbubble supply rate in the terminal arterioles for the baseline and SHI networks. Note: the logarithmic scale used for the y‐axes in panels A, B, and D

**Figure 9:**
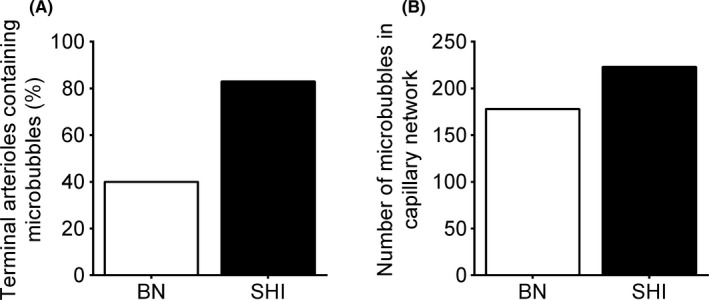
The figure depicts the mathematical modeling results as a percentage (%) of terminal arterioles containing microbubbles (A) and the number of microbubbles in the capillary network (B) at baseline and in the simulated hyperinsulinemia networks based on a theoretical network of 214 terminal arterioles and the supplied capillaries

All arterioles have RBC flow under both simulated conditions (Figure 7A,B). In contrast, at baseline, microbubble flow is zero in the majority of terminal arterioles and a large number of higher order arterioles as well (Figure 7C). Although it is challenging to perceive the subtle redistribution of RBC flow in the simulated hyperinsulinemia network (comparing Figure 7A,B), the redistribution of microbubble flow in terminal arterioles is clear (comparing Figure 7C,D). In Figure 7D, only a few branches have zero microbubble flow under the simulated hyperinsulinemia conditions. Figure 8A, 8, and 8 show the distribution of RBC flow, plasma flow and hematocrit in the 214 terminal arterioles under both simulated conditions. As expected, the reduced heterogeneity of arteriolar resistances and increased driving pressure across the network in the simulated hyperinsulinemia case were associated with an increase in both RBC and plasma flow with a small reduction in the range of values (Figure 8A, 8). Interestingly, the simulated redistribution of flow was not sufficient to cause a substantial change in terminal arteriolar hematocrit, which had a mean of 29% in both cases; however, there was a reduction in the heterogeneity of hematocrit values between terminal arterioles (Figure 8C). The redistribution of microbubble flow observed in Figure 7C,D is clearly demonstrated in Figure 8D. At baseline conditions, microbubble flow in terminal arterioles was extremely heterogeneous; 60% had no microbubbles (Figure 9A) while 4.7% carried more than one microbubble s^−1^ (Figure 8D). Under simulated hyperinsulinemic conditions, the distribution of microbubble flow was more uniform among the terminal arterioles resulting in only 17% with no microbubbles (Figure 9A). Of the terminal arterioles with microbubble flow, none carried more than one microbubble s^−1^.

The percentage of terminal arterioles with microbubble flow increased by 107% in the simulated hyperinsulinemia network (Figure 9A). Despite the increase in terminal arterioles receiving microbubbles, our microbubble distribution model predicts that the total number of microbubbles in the entire arteriolar network decreased slightly from 31.5 in the baseline network to 30.1 in the simulated hyperinsulinemia network. However, with microbubbles distributed to more terminal arterioles, the total number of microbubbles in capillaries increased from 178 microbubbles at baseline to 223 microbubbles, under the simulated hyperinsulinemia conditions (Figure 9B). Thus, the modeled flow change results in a 25% increase in microbubbles in the capillary bed with a negligible change in microvascular blood volume (<1% due to diameter changes in the simulated hyperinsulinemia arteriolar network) and no capillary recruitment.

## 4 DISCUSSION

In this study, hyperinsulinemia at a constant glucose concentration resulted in an increase in mean erythrocyte and plasma flow in the microvasculature of the rat EDL muscle. This occurred without evidence of de novo capillary recruitment either within individual capillary networks or among networks supplied by different terminal arterioles. However, hyperinsulinemia was associated with a redistribution of RBC flow toward a more homogenous erythrocyte distribution. There was no evidence of a redistribution of plasma flow. Our computational model of microvasculature flow predicts that a more homogeneous flow distribution would result in a more homogeneous distribution of microbubbles in the arteriolar tree and hence an increased number of microbubbles distributed more uniformly across capillary networks. This would suggest that IVVM and CEU observations are not in conflict if the increase in CEU signal was interpreted as representing a more uniform distribution of microbubbles in capillaries with pre‐existing erythrocyte and plasma flow. There is no fundamental reason for assuming the increase in CEU signal must be due to de novo capillary recruitment.

### 4.1 Redistribution of erythrocyte flow but not plasma flow

There are numerous publications reporting that insulin plays a significant role in regulating the distribution of microvascular blood flow to enhance the delivery of both insulin and glucose to skeletal muscle.^2^, ^3^, ^5^, ^6^, ^7^ Our experiments, using IVVM to quantify microvascular blood flow in the rat EDL muscle, confirm that an increased insulin concentration while infusing glucose to maintain a fixed blood glucose level does result in increased RBC velocity, RBC supply rate, and RBC flow (Figures 2A and 3F,A; but in contrast to previous reports using indirect methods,^2^, ^3^, ^4^, ^5^, ^6^, ^7^ we found no evidence of de novo capillary recruitment, either within individual capillary networks (Figure 4) or among networks supplied by different terminal arterioles (Figure 5). However, we did find a redistribution of RBC flow as shown in Figure 6A. The significant negative slope for the change in RBC flow from baseline vs hyperinsulinemia indicates a redistribution of RBC flow from high to low flow capillaries with hyperinsulinemia. In contrast, the slope for plasma flow in these same capillaries (Figure 6B) was not significantly different from zero. Although hyperinsulinemia did increase plasma flow (Figure 3B), we saw no evidence for a controlled redistribution of insulin and glucose as has been proposed.^47^

Our observation that the distribution of plasma might be affected differently by hyperinsulinemia than erythrocyte distribution is not surprising. Plasma as a Newtonian fluid distributes proportionally to the downstream hemodynamic resistance. Erythrocytes, as highly deformable cells, demonstrate important rheological properties in the microvasculature as the size of the cell approaches the diameter of the vessel. Erythrocytes migrate away from regions of high shear rate near the vessel wall toward the centerline^48^, ^49^ causing a radial gradient in erythrocyte concentration. As a consequence, although the plasma carries erythrocytes, on average erythrocytes travel faster than plasma (Fahreaus Effect)^36^ and are preferentially distributed at bifurcations to the branch with the higher plasma flow. Small changes in erythrocyte distribution at bifurcations have a cascading effect across multiple branches of the arteriolar tree resulting in mean capillary hematocrit approximately half of systemic hematocrit. In this study mean, capillary hematocrit, measured using IVVM, was 17% compared with systemic hematocrit of 37.5% (Figure 2B & Table 1). The network computational model also demonstrated a drop in hematocrit from the largest arteriole to the terminal arteriole of 35% to 29%. Not all capillaries can have low hematocrit, as demonstrated in our computational model for terminal arterioles (Figure 8C). A small number of flow paths (those with the higher flow at each bifurcation) are preferentially enriched with erythrocytes as indicated in Figures 7A and 8A, reaching values in the terminal vessels near or above the hematocrit in the largest arteriole. Although plasma velocity is slightly less than RBC velocity in capillaries, the low capillary hematocrit results in plasma flow being approximately fourfold higher than erythrocyte flow at baseline and threefold higher during the hyperinsulinemia euglycemic clamp.

In summary, our IVVM data yield a very different interpretation of the effect of insulin on microvascular blood flow than has been previously presented by others based on CEU data. During the hyperinsulinemia euglycemic clamp, there was a substantial increase in plasma flow without evidence of de novo capillary recruitment or redistribution of plasma flow. However, there was evidence for both an increase in erythrocyte flow and a more uniform distribution of erythrocyte flow within the capillary bed. Whether this redistribution of erythrocyte flow has a functional consequence for glucose uptake or is in response to altered oxygen requirements within the tissue is unknown.

### 4.2 Computational model shows increased microbubble numbers with flow redistribution

Our computational model addressed the question of whether a redistribution of microbubbles within the microvasculature without de novo capillary recruitment could result in an increased CEU signal. Experiments show that an increased flow rate such as we measured in capillaries does not increase the CEU ultrasound signal, whereas a redistribution of microbubbles to a larger number of flow paths does.^50^ The model shows that a few terminal arterioles corresponding to the paths with the highest flow rate have hematocrits approaching systemic hematocrit (Figure 8C and Table 1). At the low microbubble density of 1:6000 used in human studies 4 and assuming microbubbles have the same bifurcation law as erythrocytes, microbubbles are mostly found in these high flow paths. Increasing the flow does not affect the number of microbubbles in the capillary bed (more microbubbles enter but with higher velocities they have shorter residence times) in agreement with Ross et al.^50^ Reducing the asymmetry of flow does increase the number of terminal arterioles with microbubbles but it does not increase the total number of microbubbles resident within the capillary bed itself (model results not shown). If, however, the computational model uses a bifurcation rule with microbubbles being more preferentially distributed at bifurcations than erythrocytes, the result is an enrichment of microbubbles relative to erythrocytes in the high flow paths. This enrichment of microbubbles is consistent with Lindner et al’s observation of microbubbles in capillaries being separated by many fewer RBCs than one would expect.^27^ Under these conditions, a reduction in flow asymmetry distributes more microbubbles into more terminal arterioles which results in an increase in the total number of microbubbles in the capillary bed. In an actual experiment, this increase in total microbubble number would be observed as an increased CEU signal and hence interpreted as an increased microvascular volume and evidence of capillary recruitment. However in the computational model, the microbubbles are redistributed, not into newly recruited capillaries, but into capillaries which are already perfused with erythrocytes and plasma.

Thus, the computational model introduces a new interpretation of CEU data that is consistent with our IVVM results. The increased CEU signal with hyperinsulinemia reflects a reduction in the asymmetry of erythrocyte flow and a “recruitment” of capillaries carrying microbubbles. It does not reflect an increase in microvascular volume due to de novo recruitment of “nutritive” capillaries carrying erythrocytes and plasma. Since the relatively small number of microbubbles are most likely limited to the highest arteriolar flow paths, their redistribution with insulin would primarily provide insight on these higher flow paths while yielding limited insight into glucose uptake from the vast majority of capillaries in the muscle.

### 4.3 Evidence that EDL preparation reflects normal physiology

Proponents of capillary recruitment have criticized IVVM muscle preparations as not reflecting normal physiological function. They argue that surgery to expose these thin muscles likely result in hyperemia thus masking the capillary recruitment which one would observe using non‐invasive techniques such as CEU on thicker muscles which play a more important role in physical activity.^8^, ^9^ To address these potentials criticisms, we used the rat EDL muscle, a bellied muscle with mixed fiber type, which is involved in locomotion by controlling the movement of the hind paw. Tyml^19^ developed this IVVM microvascular preparation because it required minimal surgery with no evidence of trauma and it could be studied without the use of a superfusion solution. They demonstrated that contraction of the muscle resulted in a tenfold increase in RBC velocity without an increase in perfused capillary density.^19^ Although our preparation of the EDL for transillumination requires tying a suture around the distal tendon and cutting the tendon between the suture and its attachment, the muscle maintains a stable microvascular perfusion with resting RBC velocities for up to 5‐6 hours^13^, ^33^, ^51^, ^52^ similar to those reported by Tyml.^19^ Two independent research groups have also shown that exteriorization does not alter muscle flow.^25^, ^26^ Further evidence that our EDL preparation reflects the normal physiological response comes from the 80% increase in RBC supply rate (Figure 3F) and corresponding increase in RBC O_2_ saturation from 50 ± 3 to 67 ± 11% (Figure 3E) with the insulin stimulus. If microvascular blood flow had been hyperemic with abnormally high capillary recruitment under resting conditions, the baseline O_2_ saturations would have been much higher and the modest increase in flow with insulin should have had little effect on O_2_ saturations values. The increase in O_2_ saturation with insulin is consistent with the increase in RBC supply rate under conditions with constant capillary density and O_2_ consumption.^53^

Thus, these results also confirm the overwhelming consensus of previous IVVM studies that de novo capillary recruitment does not occur as a normal physiological response.^54^ However, redistribution of blood flow within capillary networks is observed with IVVM that does not involve the opening of new capillary flow paths. This redistribution results in a more uniform distribution of RBCs among perfused capillaries and reduces the heterogeneity of capillary hematocrits and supply rates.^24^ Less heterogeneity of RBC supply rate is sufficient to maintain tissue oxygenation as O_2_ consumption rates increase,^55^, ^56^ without the need for increased capillary density. De‐recruitment of perfused capillaries also occurs if the IVVM preparation is exposed to elevated O_2_ levels^57^ or to pathological conditions such as sepsis.^58^

The baseline RBC O_2_ saturation of 50 ± 3% and RBC supply rate of 6 ± 1 RBCs s^−1^ were lower than we have previously reported^13^ for capillaries of the rat EDL muscle. In previous studies, the rats were anesthetized with pentobarbital which suppresses ventilation^59^ and hence were ventilated with 30% inspired O_2_ to maintain high arterial pO_2_ levels and arterial hemoglobin oxygen saturation above 95%. In the current study, the animals were anesthetized with α‐chloralose/urethane to avoid suppression of autonomic, cardiovascular, and respiratory function.^59^, ^60^ Since the animals were breathing room air spontaneously, they had lower arterial pO_2_ (78 ± 4 mm Hg) and hemoglobin oxygen saturation (91 ± 1%) levels, which would affect the baseline capillary O_2_ saturations. The choice of anesthetic may also account for the lower capillary RBC supply rate due to increased sympathetic nervous system activity and arteriolar vasoconstriction, which would also contribute to the lower capillary O_2_ saturations at baseline.

Overall, this quantitative microvascular data provide evidence that the flow response to hyperinsulinemia measured in the rat EDL muscle reflects a normal physiological response to insulin.

## 5 CONCLUSION

Hyperinsulinemia under euglycemic conditions results in an increase in mean erythrocyte and plasma flow in the microvasculature of the rat EDL muscle without evidence of de novo capillary recruitment either within individual capillary networks or among networks supplied by different terminal arterioles. Hyperinsulinemia results in more homogeneous distribution of RBC flow, but there is no evidence of a redistribution of plasma flow and hence no evidence that insulin regulates its own distribution (vs overall supply) and that of glucose.

Our computational model of RBC, plasma, and microbubble distribution predicts that a more homogeneous RBC flow distribution is associated with a higher number of microbubbles in capillary networks without the need for de novo capillary recruitment. A higher number of microbubbles would translate into an increase in CEU signal, thus suggesting that divergent findings using CEU and IVVM can be reconciled.

## PERSPECTIVE

Hyperinsulinaemia under euglycaemic conditions results in an increase in mean blood flow and a more homogeneous distribution of red blood cell flow without evidence of a redistribution of plasma flow or de novo capillary recruitment. Our computational model demonstrates that this redistribution of RBC flow would account for the increased signal associated with CEU studies following insulin administration and hence suggest a reinterpretation of CEU data. This study highlights that IVVM is a powerful tool for studying how the microvasculature redistributes blood flow in response to a stimulus, change in tissue demand, or potentially how it may fail in acute or chronic disease.

## CONFLICT OF INTEREST

We have no competing interests to declare.

## AUTHORS' CONTRIBUTIONS

TA, FN, CGE and SLM designed the study, TA, FN, CGE and SLM carried out the experiments, and DG performed the mathematical modeling. CLB and GMF contributed with data. TA, CGE, GMF and DG wrote the paper. All authors contributed to, and approved, the final version of the manuscript. The experiments were conducted at the Department of Medical Biophysics, Schulich School of Medicine & Dentistry, University of Western Ontario, London, Canada.

## APPENDIX 1

### Microbubble flow model

There are two related properties of microbubble flow in microvascular networks that affect their network dynamics: the mean velocity in individual vessel segments and the relative distribution at diverging bifurcations. Knowing these properties, our blood flow model^39^, ^61^ can be applied to determine microbubble flow and density throughout a given microvascular network, assuming the number of microbubbles per unit blood volume is always low enough (eg, ≤1/6000 the number of RBCs as in CEU experiments^62^ to assure that the microbubbles will not have an appreciable effect on blood rheology (viscosity or RBC flow properties).

If volume and flow‐averaged microbubble volume densities are denoted ϕ*_T_* and ϕ*_D_*, respectively, the microbubble conservation equation at any node is:

(1) $$\sum_{i}\phi_{D,i}Q_{i}=0$$

where the sum is over all vessel segments *i* connected to the node being considered, *Q_i_* is blood flow rate in segment *i*, and the microbubble flow rate is $Q_{M,B,i}=\phi_{D,i}Q_{i}$. The two microbubble flow properties mentioned above are analogous to the Fahraeus effect and bifurcation law for RBCs. The microbubble Fahraeus effect gives the relation between ϕ*_T_* and ϕ*_D_*_,_ which are connected by the equation $v_{\mathrm{MB}}\phi_{T}=v_{\text{blood}}\phi_{D}$ where *v_MB_* is the radially averaged microbubble velocity.

For arteriolar diameter D > 30 microns, we approximate the Fahraeus effect and bifurcation law for microbubbles by assuming they are the same as for RBCs, based on the measurements in Keller et al.^28^ For D < 30 microns, where experimental data are fairly sparse, the bifurcation law is altered if the flow ratio is greater than 2 (higher daughter flow divided by lower daughter flow), in which case all microbubbles enter the high‐flow branch. This simple model allows us to explore the possible effects of microbubble distribution at diverging bifurcations being more preferential than RBC distribution.

### Capillary microbubble model

We assume each terminal arteriole supplies the same number of capillaries (*N_c_ = 103*) and that each capillary supplied by a given terminal arteriole receives the same blood flow (*Q*) and microbubble supply rate (*SR_MB_*). For a capillary with radius *R_c_* (=2.5 microns), the blood and microbubble velocity are estimated as $v_{\text{blood}}=v_{\mathrm{MB}}=Q/\left(\pi \cdot R_{c}^{2}\right)$. The lineal density of microbubbles is then *LD_MB_ = SR_MB_/v_MB_*, and the number of resident microbubbles is *N_MB_ = L_c_⋅LD_MB_* where *L_c_* (=500microns) is the capillary length. The total number of resident microbubbles in capillaries supplied by the given terminal arteriole is then *Nc N_MB._*
