# Supplementary material for: Hyperinsulinemia does not cause de novo capillary recruitment in rat skeletal muscle
Source: Microcirculation. 2019 Oct 12;27(2):e12593. doi: 10.1111/micc.12593 (PMC7064932; doi:10.1111/micc.12593)
Supplement: Supplementary file 1 [file MICC-27-e12593-s001.docx]

**Supporting information**

The supplementary material show two video recordings of microvascular blood flow in the rat EDL muscle at 10X magnification taken using the 442-nm interference filter. The 442-nm video was chosen because of the greater contrast between the RBCs and surrounding tissue. The first video was acquired during baseline infusion of saline and the second video of the same FOV was acquired 20 minutes following initiation of the hyperinsulinaemic euglycaemic clamp protocol. The FOV is oriented with the muscle fibers running from top to bottom of the image. Capillaries with single file RBC flow run parallel to the muscle fibers with some cross-branches forming networks of capillary segments supplied by terminal arterioles and draining into collecting venules. Terminal arterioles and venules arise from deeper in the tissue and for the most part run obliquely across the muscle fibers. The videos demonstrate that almost all capillaries carrying RBC flow 20 minutes into the hyperinsulinaemic euglycaemic clamp were also filled with RBC flow at baseline. There is no evidence of *de novo* capillary recruitment.
